# A High-Resolution and Low-Complexity DOA Estimation Method with Unfolded Coprime Linear Arrays

**DOI:** 10.3390/s20010218

**Published:** 2019-12-30

**Authors:** Wei He, Xiao Yang, Yide Wang

**Affiliations:** 1 School of Information Engineering, Guangdong University of Technology, Guangzhou 510006, China; hwjy2099@163.com; 2 Institute d’Electronique et Télécommunications de Rennes (IETR), Université de Nantes, 44306 Nantes, France; yide.wang@univ-nantes.fr

**Keywords:** DOA estimation, unfolded coprime linear array, Toeplitz matrix, high resolution, low complexity

## Abstract

The direction-of-arrivals (DOA) estimation with an unfolded coprime linear array (UCLA) has been investigated because of its large aperture and full degrees of freedom (DOFs). The existing method suffers from low resolution and high computational complexity due to the loss of the uniform property and the step of exhaustive peak searching. In this paper, an improved DOA estimation method for a UCLA is proposed. To exploit the uniform property of the subarrays, the diagonal elements of the two self-covariance matrices are averaged to enhance the accuracy of the estimated covariance matrices and therefore the estimation performance. Besides, instead of the exhaustive peak searching, the polynomial roots finding method is used to reduce the complexity. Compared with the existing method, the proposed method can achieve higher resolution and better estimation performance with lower computational complexity.

## 1. Introduction

Direction-of-arrival (DOA) estimation is one of the most active research topics in the field of array signal processing, and it has been widely used in radar, sonar, radio astronomy and other fields [1,2,3,4]. Many DOA estimation methods, such as Multiple Signal Classification (MUSIC) [5] and Estimation of Signal Parameters via Rotational Invariance Techniques (ESPRIT) [6], have been well developed for uniform linear arrays (ULAs), in which the inter-element spacing is restricted to the half-wavelength of incoming signals, leading to a possible mutual coupling effect and inferior estimation performance. To solve these problems, coprime linear arrays (CLAs) have been proposed in [7]. Composed of two ULAs with larger inter-element spacing, a CLA can achieve a larger array aperture with less mutual coupling effect, and consequently better effective estimation performance can be obtained.

The research of the DOA estimation with a CLA can be classified into two categories, which are difference-coarray-based methods and subarray-based methods. The difference-coarray-based methods aim to increase the degrees of freedom (DOFs). However, this class of methods requires a great number of snapshots, which makes it computationally complex [8,9,10,11]. In the subarray-based methods, a CLA is treated as two sparse uniform subarrays. Separately dealing with them, the uniform property of the two subarrays can be directly exploited, making low-complexity DOA estimation possible. Besides, the true DOAs can be determined based on the coinciding results of the two subarrays, and the ambiguities caused by the large inter-element spacing can be avoided thanks to the coprime property [12,13,14]. As they are more suitable for practical applications, many subarrays-based methods have been proposed in recent years. In [12], the MUSIC algorithm is performed separately in both subarrays, and the true DOAs are determined by finding the coinciding peaks of the two spectrums. In [13], the above-mentioned method is improved; by taking advantage of the properties of complex exponential functions, the method limits the peak-searching region and reduces the computational complexity. Another method is proposed in [14]. Benefitting from the uniform property of the two subarrays, ESPRIT is employed. Without the step of peak-searching, the complexity is significantly reduced. Besides, the potential matching error problem in [12,13] is fixed with beamforming-based methods.

Unfortunately, dealing with the two subarrays separately, the subarray-based methods have the following problems: (i) the DOF is determined by the subarray with fewer sensor elements, and therefore the number of detectable signals is limited; (ii) the mutual information of the two subarrays is lost, resulting in an inferior estimation performance; and (iii) an additional step is needed to pair the results obtained from the two subarrays, which increases the computational complexity.

In order to solve these problems, a method based on a new geometry of unfolded coprime linear arrays (UCLAs) is proposed in [15]. By rotating a subarray of a CLA 180°, a non-uniform linear array with a larger aperture can be obtained. Instead of treating the two subarrays separately, MUSIC is performed on the outputs of the whole array. Being superior to a CLA and subarray-based methods, the UCLA can achieve full DOFs as well as better estimation performance due to the exploitation of both self and mutual information. Meanwhile, thanks to the coprime property, only the peaks associated with the true DOAs exist in the MUSIC spectrum. However, this method still has some problems: (i) the uniform property of the two subarrays is wasted; and (ii) the step of peak-searching is involved, increasing the computational complexity.

In this paper, an improved DOA estimation method is proposed. Taking advantage of the uniform property of the two subarrays, we average the diagonal elements of the estimated self-covariance matrices of the observation of the two subarrays to make the covariance matrix of the outputs of the whole array partially Toeplitz. Besides, instead of traditional MUSIC, root-MUSIC is used to avoid the step of peak-searching. Compared with the method in [15], the proposed method can achieve higher resolution and better estimation performance with lower computational complexity. Simulation results are provided to show the performance of the proposed method.

## 2. System Model

**Figure 1:**
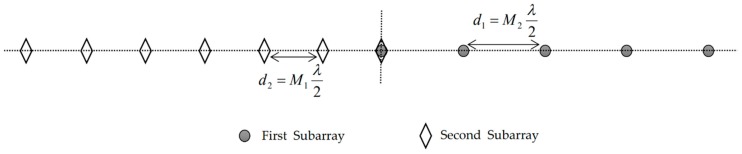
System model.

Consider a UCLA composed of two uniform linear subarrays, in which the numbers of sensors are $M_{1}$ and $M_{2}$, and the inter-element spacings are $d_{1}=M_{2}\lambda /2$ and $d_{2}=M_{1}\lambda /2$, respectively, with $M_{1}$ and $M_{2}$ being two coprime integers and λ the wavelength of incoming signals. One sensor element is shared by the two subarrays and is set as the reference point. The total number of sensors $M=M_{1}+M_{2}-1$. Figure 1 shows the case of $M_{1}=5$ and $M_{2}=7$.

Assume that K (K is supposed to be known or can be correctly estimated by the Akaike Information Criterion (AIC) or Minimum Description Length (MDL) method [16], and K<M) uncorrelated, far-field and narrowband signals impinge on the UCLA from directions $\left\{\theta_{1},\;\theta_{2},\ldots ,\theta_{K}\right\}$, with $\theta_{k}\in \left(-\pi /2,\pi /2\right)$ and k∈[1, K]. The signals received at the two subarrays can be respectively written as

(1) $$x_{1}\left(t\right)=A_{1}s\left(t\right)+n_{1}\left(t\right)$$

(2) $$x_{2}\left(t\right)=A_{2}s\left(t\right)+n_{2}\left(t\right)$$

where $A_{i}=\left[\begin{array}{cccc} a_{i}\left(\theta_{1}\right) & a_{i}\left(\theta_{2}\right) & \cdots & a_{i}\left(\theta_{K}\right) \end{array}\right]$, (i=1, 2) is the directional matrix with

(3) $$a_{1}\left(\theta_{k}\right)={\left[\begin{array}{cccc} 1 & e^{jM_{2}\pi \sin\left(\theta_{k}\right)} & \cdots & e^{j\left(M_{1}-1\right)M_{2}\pi \sin\left(\theta_{k}\right)} \end{array}\right]}^{T}$$

(4) $$a_{2}\left(\theta_{k}\right)={\left[\begin{array}{cccc} e^{-j\left(M_{2}-1\right)M_{1}\pi \sin\left(\theta_{k}\right)} & e^{-j\left(M_{2}-2\right)M_{1}\pi \sin\left(\theta_{k}\right)} & \cdots & 1 \end{array}\right]}^{T}$$

which are the directional vectors of the two subarrays, respectively. $s\left(t\right)\in {\mathbb{C}}^{K\times 1}$ denotes the source vector, and $n_{i}\left(t\right)\in {\mathbb{C}}^{M_{i}\times 1}$ the white Gaussian noise vector with zero-mean and covariance matrix $\sigma^{2}I_{M_{i}}$, which is independent from the source signals. Therefore, the total outputs of the UCLA can be written as

(5) $$x\left(t\right)=\left[\begin{array}{c} x_{1}\left(t\right)\\ x_{2}\left(t\right) \end{array}\right]=\left[\begin{array}{c} A_{1}\\ A_{2} \end{array}\right]s\left(t\right)+\left[\begin{array}{c} n_{1}\left(t\right)\\ n_{2}\left(t\right) \end{array}\right]$$

## 3. Proposed Method

### 3.1. Self-Covariance Matrix Reconstruction

The covariance matrix of the total output of the UCLA R can be estimated with L snapshots as

(6) $$\hat{R}=\frac{1}{L}\overset{L}{\underset{t=1}{\sum }}x\left(t\right)x^{H}\left(t\right)=\left[\begin{array}{cc} {\hat{R}}_{11} & {\hat{R}}_{12}\\ {\hat{R}}_{21} & {\hat{R}}_{22} \end{array}\right]$$

with ${\hat{R}}_{uv}=\frac{1}{L}\sum_{t=1}^{L}x_{u}\left(t\right)x_{v}^{H}\left(t\right)$ and u,v∈{1,2}.

The self-covariance matrix of the observation of either subarray can be written as

(7) $$R_{uu}=\left[\begin{array}{cccc} r_{u}\left(0\right) & r_{u}\left(-1\right) & \cdots & r_{u}\left(1-M_{u}\right)\\ r_{u}\left(1\right) & r_{u}\left(0\right) & \cdots & r_{u}\left(2-M_{u}\right)\\ \vdots & \vdots & \ddots & \vdots \\ r_{u}\left(M_{u}-1\right) & r_{u}\left(M_{u}-2\right) & \cdots & r_{u}\left(0\right) \end{array}\right]$$

where $r_{u}\left(m\right)=\sum_{k=1}^{K}P_{s_{k}}e^{jmM_{v}\sin\left(\theta_{k}\right)}$, u≠v, and $r_{u}\left(-m\right)=r_{u}^{*}\left(m\right)$. $P_{s_{k}}$ denotes the power of the signal coming from direction $\theta_{k}$.

It can be observed that, because both the subarrays are uniform, the self-covariance matrix of the signal received at either subarray is Toeplitz. All the diagonal elements are equal, and this can be uniquely determined by its first column of the self-covariance matrix. To exploit this property, we can average the diagonal elements of the estimated self-covariance matrix ${\hat{R}}_{uu}$ by

(8) $${\hat{r}}_{u}\left(m\right)=\frac{1}{M_{u}-m}\sum_{n=1}^{M_{u}-m}{\hat{R}}_{uu}\left(m+n,n\right)$$

According to Equation (7), depending on the two sets of averaged elements $\left\{{\hat{r}}_{1}\left(0\right),{\hat{r}}_{1}\left(1\right),\cdots ,{\hat{r}}_{1}\left(M_{1}-1\right)\right\}$ and $\left\{{\hat{r}}_{2}\left(0\right),{\hat{r}}_{2}\left(1\right),\cdots ,{\hat{r}}_{2}\left(M_{2}-1\right)\right\}$, two Toeplitz self-covariance matrices ${\hat{R}}_{11,T}$ and ${\hat{R}}_{22,T}$ can be constructed. Replacing ${\hat{R}}_{11}$ and ${\hat{R}}_{22}$ by ${\hat{R}}_{11,T}$ and ${\hat{R}}_{22,T}$ in $\hat{R}$, respectively, we can make the covariance matrix of the total outputs of the UCLA partially Toeplitz, which can improve the estimate performance. In contrast with the traditional covariance matrix averaging technique, which is performed on the whole covariance matrix of the received signals, the proposed partial Toeplitz averaging method is particularly well adapted to unfolded coprime linear arrays. The reconstructed partial Toeplitz covariance matrix ${\hat{R}}_{T}$ is given as

(9) $${\hat{R}}_{T}=\left[\begin{array}{cc} {\hat{R}}_{11,T} & {\hat{R}}_{12}\\ {\hat{R}}_{21} & {\hat{R}}_{22,T} \end{array}\right]$$

### 3.2. DOA Estimation

After the reconstruction of the covariance matrix, the eigenvalue decomposition of the obtained partial Toeplitz matrix ${\hat{R}}_{T}$ can be expressed as

(10) $${\hat{R}}_{T}={\hat{U}}_{s}{\hat{\Lambda }}_{s}{\hat{U}}_{s}^{H}+{\hat{U}}_{n}{\hat{\Lambda }}_{n}{\hat{U}}_{n}^{H}$$

where ${\hat{U}}_{s}$ contains the eigenvectors spanning the signal subspace and ${\hat{\Lambda }}_{s}$ a diagonal matrix composed of the K largest eigenvalues of ${\hat{R}}_{T}$, the eigenvalue matrix corresponding to ${\hat{U}}_{s}$; ${\hat{U}}_{n}$ contains the eigenvectors spanning the noise subspace and ${\hat{\Lambda }}_{n}$ the eigenvalue matrix corresponding to ${\hat{U}}_{n}$. According to the orthogonality between the signal and noise subspaces, the spectrum function can be written as

(11) $$P\left(\theta \right)=\frac{1}{\left[\begin{array}{cc} a_{1}^{H}\left(\theta \right) & a_{2}^{H}\left(\theta \right) \end{array}\right]{\hat{U}}_{n}{\hat{U}}_{n}^{H}{\left[\begin{array}{cc} a_{1}^{T}\left(\theta \right) & a_{2}^{T}\left(\theta \right) \end{array}\right]}^{T}}$$

and the DOAs can be found by searching the peaks of the spectrum P(θ).

To reduce the computational complexity, the polynomial root finding method can be used instead of an exhaustive search. Define

(12) $$p\left(z\right)={\left[\begin{array}{cc} p_{1}^{T}\left(z\right) & p_{2}^{T}\left(z\right) \end{array}\right]}^{T}$$

with

(13) $$p_{1}\left(z\right)={\left[\begin{array}{cccc} 1 & z^{M_{2}} & \cdots & z^{\left(M_{1}-1\right)M_{2}} \end{array}\right]}^{T}$$

(14) $$p_{2}\left(z\right)={\left[\begin{array}{cccc} z^{-\left(M_{2}-1\right)M_{1}} & z^{-\left(M_{2}-1\right)M_{1}} & \cdots & 1 \end{array}\right]}^{T}$$

which are related to the directional vectors of the two subarrays by

(15) $$a_{1}\left(\theta \right)=p_{1}\left(z=e^{j\pi \sin\left(\theta \right)}\right)$$

(16) $$a_{2}\left(\theta \right)=p_{2}\left(z=e^{j\pi \sin\left(\theta \right)}\right)$$

Then, the exhaustive peak search in Equation (11) can be transformed to the root finding of the following polynomial:

(17) $$p^{T}\left(z^{-1}\right){\hat{U}}_{n}{\hat{U}}_{n}^{H}p\left(z\right)=0$$

It can be seen that, since the sensor elements are sparsely and non-uniformly located, p(z) contains only several discrete powers of z and Equation (17) is not a full polynomial. In order to solve this problem, we define two transformation matrices as

(18) $$G_{1}={\left[\begin{array}{cc} 0_{1} & H_{1} \end{array}\right]}_{M_{1}\times \left(2M_{1}M_{2}-M_{1}-M_{2}+1\right)}$$

(19) $$G_{2}={\left[\begin{array}{cc} H_{2} & 0_{2} \end{array}\right]}_{M_{2}\times \left(2M_{1}M_{2}-M_{1}-M_{2}+1\right)}$$

where $H_{1}$ is a selection matrix with a dimension of $M_{1}\times \left[\left(M_{1}-1\right)M_{2}+1\right]$, of which the $\left[\left(i-1\right)M_{2}+1\right]\mathrm{th}$ element of the ith row is one and the other elements are zeros; $H_{2}$ is a selection matrix with the dimension of $M_{2}\times \left[\left(M_{2}-1\right)M_{1}+1\right]$, of which the $\left[\left(i-1\right)M_{1}+1\right]\mathrm{th}$ element of the $i^{\mathrm{th}}$ row is one and the other elements are zeros. $0_{1}$ and $0_{2}$ are two zero matrices with the dimension of $M_{1}\times \left(M_{2}-1\right)M_{1}$ and $M_{2}\times \left(M_{1}-1\right)M_{2}$, respectively.

Then, we get

(20) $$p\left(z\right)=\left[\begin{array}{c} G_{1}\\ G_{2} \end{array}\right]p_{u}\left(z\right)$$

where

(21) $$p_{u}\left(z\right)={\left[\begin{array}{ccccccc} z^{-\left(M_{2}-1\right)M_{1}} & z^{-\left(M_{2}-1\right)M_{1}+1} & \cdots & 1 & \cdots & z^{\left(M_{1}-1\right)M_{2}-1} & z^{\left(M_{1}-1\right)M_{2}} \end{array}\right]}^{T}$$

which contains all continuous power of z. Therefore, Equation (17) can be transformed as

(22) $$p_{u}^{T}\left(z^{-1}\right)U_{nn}U_{nn}^{H}p_{u}\left(z\right)=0$$

where

(23) $$U_{nn}=\left[\begin{array}{cc} G_{1}^{T} & G_{2}^{T} \end{array}\right]{\hat{U}}_{n}$$

Equation (22) is a full polynomial to which the root finding technique can be directly applied. It is known that if $\theta_{i}$ corresponds to the direction of an actual source, $z_{i}=e^{j\pi \sin\left(\theta_{i}\right)}$ would be a root of Equation (22), and $\left|z_{i}\right|=\left|e^{j\pi \sin\left(\theta_{i}\right)}\right|=1$. However, because of the presence of noise, the roots may not be precisely located on the unit circle. Besides, note that if $z_{i}$ is a root of Equation (22), so is $1/z_{i}^{*}$. Therefore, half of the roots will be inside the unit circle and half will be outside. The DOAs can be decided by the K roots inside and closest to the unit circle as

(24) $${\hat{\theta }}_{k}=\arcsin\left(\frac{\arg\left({\hat{z}}_{k}\right)}{\pi }\right)$$

## 4. Simulation and Analysis

### 4.1. Estimation Performance

In the simulations, the UCLA shown in Figure 1 with $M_{1}=5$ and $M_{2}=7$ is considered, and the root mean square error (RMSE) is used for the performance assessment, which is defined as

(25) $$\mathrm{RMSE}=\sqrt{\left(\frac{1}{QK}\right)\sum_{q=1}^{Q}\sum_{k=1}^{K}{\left({\hat{\theta }}_{k,q}-\theta_{k}\right)}^{2}}$$

with K the number of source signals, Q the number of Monte Carlo trials, and ${\hat{\theta }}_{k,q}$ the estimate of the true DOA $\theta_{k}$ of the $q^{\mathrm{th}}$ Monte Carlo trial. Q=500 is used in this paper. The Cramér–Rao lower bound (CRB) for the unconditional model is also given as a benchmark [17].

**Figure 2:**
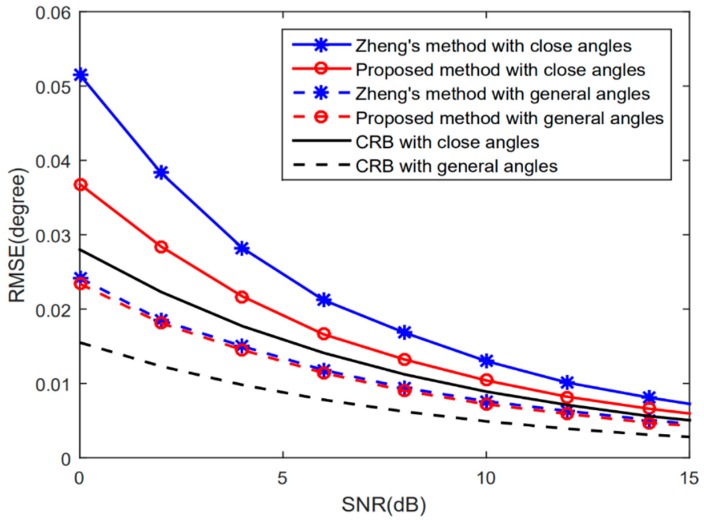
Root mean square error (RMSE) performance versus signal-to-noise ratio (SNR).

Figure 2 depicts the RMSE performance of Zheng’s method in [15] and the proposed method versus the signal-to-noise ratio (SNR) with K=2 and L=200, in both distantly separated angles situation (denoted as general angles in Figure 2), where signals come from {20°,50°}, and a close angle situation, where signals come from {24°,25°}. It can be seen that, in the distantly separated angles situation, the estimation performance is comparable to Zheng’s method; in the close angles situation, due to the reconstruction of the covariance matrix and the exploitation of the uniform property of subarrays, the proposed method has higher resolution and better estimation performance than Zheng’s method.

**Figure 3:**
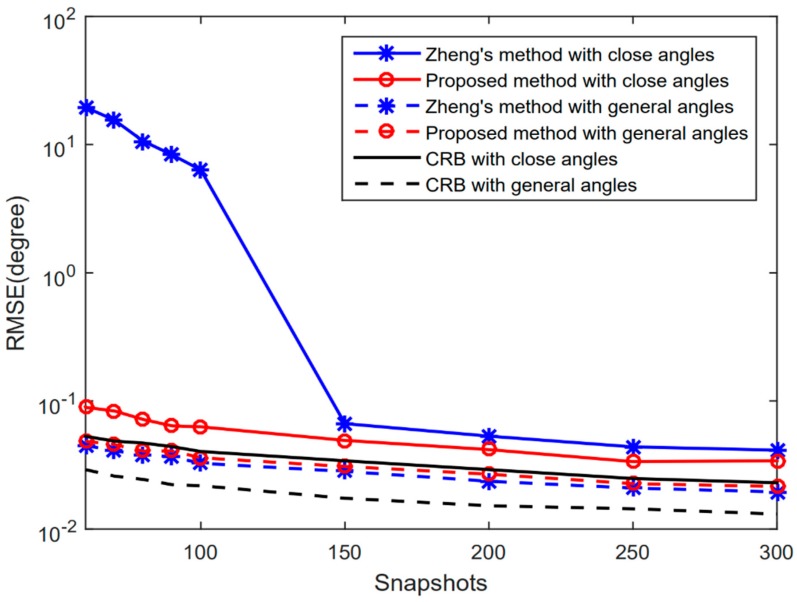
RMSE performance versus snapshots number.

Figure 3 depicts the RMSE performance of the two methods versus the snapshots number with SNR=0 dB, in both the distantly separated angles situation (denoted as general angles in Figure 3) and close angles situation. As shown in the figure, in the distantly separated situation, the two methods can achieve similar estimation performance; in the close angles situation, the performance of Zheng’s method decreases greatly for small numbers of snapshots, as MUSIC depends on the accuracy of the estimated covariance matrix. On the contrary, benefiting from the reconstructed partially Toeplitz covariance matrix, the proposed method remains robust and reliable even in the case of a small snapshots number.

**Figure 4:**
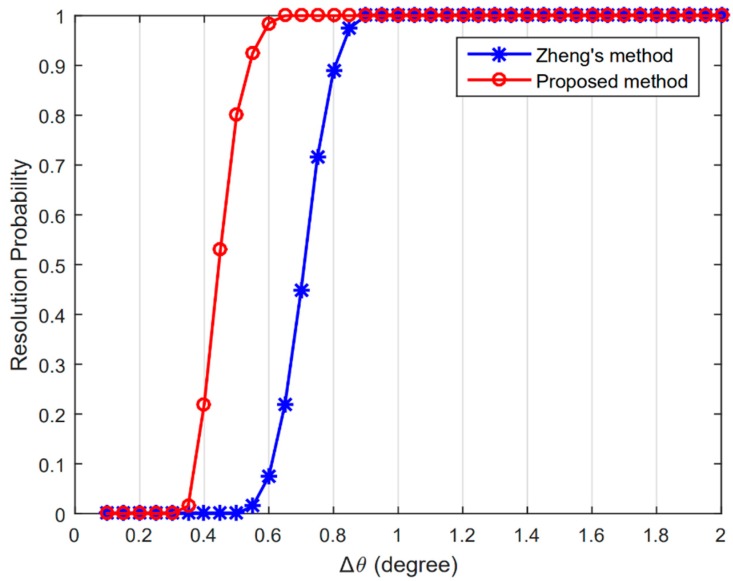
Resolution probability versus ∆θ.

To investigate the resolution of the proposed method, two signals are assumed to come from the two close directions ${∆\theta }_{1}$ and $\theta_{2}=\theta_{1}+∆\theta$, respectively, where $\theta_{1}$ is fixed at 20°, and ∆θ is a small and controllable variable. The two signals are said to be successfully resolved if the two following equations are satisfied:

(26) $$\left|{\hat{\theta }}_{1}-\theta_{1}\right|<\frac{∆\theta }{2}$$

(27) $$\left|{\hat{\theta }}_{2}-\theta_{2}\right|<\frac{∆\theta }{2}$$

where ${\hat{\theta }}_{1}$ and ${\hat{\theta }}_{2}$ are the estimations of $\theta_{1}$ and $\theta_{2}$, respectively [14,18]. Figure 4 shows the comparison of the resolution probability, which is calculated from the percentage of the success trials among 200 Monte Carlo trials, of Zheng’s method in [15] and the proposed method, with SNR=0 dB and L=200. It can be seen that, benefiting from the partial Toeplitz averaging, the accuracy of the estimated covariance matrix is enhanced, and the proposed method exhibits much better resolution performance.

### 4.2. Computational Complexity

**Figure 5:**
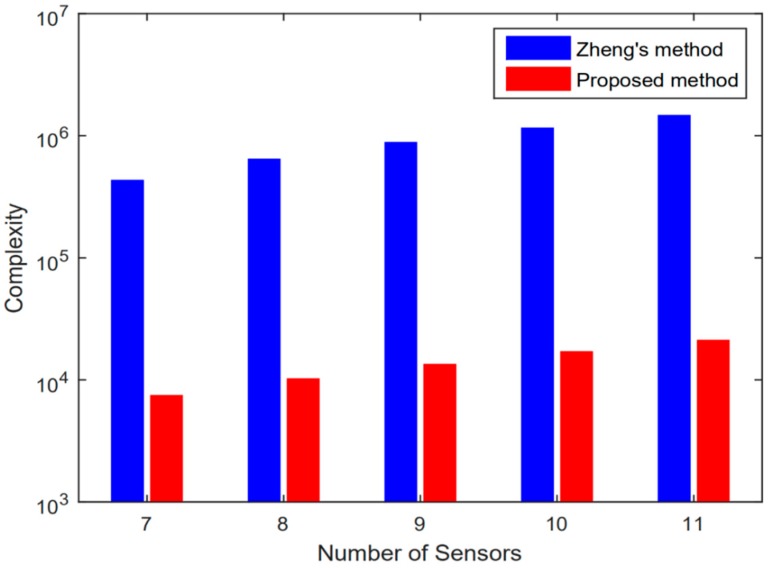
Complexity comparison.

Based on root-MUSIC, the number of complex multiplications of the proposed method is $O\left({\left(M_{1}+M_{2}\right)}^{2}L+{\left(M_{1}+M_{2}\right)}^{3}\right)$, which is obviously lower than Zheng’s method, which is $O\left({\left(M_{1}+M_{2}\right)}^{2}L+{\left(M_{1}+M_{2}\right)}^{3}+\left(M_{1}+M_{2}\right)\left(M_{1}+M_{2}-K\right)T\right)$, where L and T denote the number of snapshots and the times of spectral searching respectively. The computational complexity comparison versus the number of sensors $\left(\;M_{1}+M_{2}-1\right)$ is given in Figure 5, with K=2 and L=200. It can be seen that, without the exhaustive searching, the proposed method has much lower computational complexity with better resolution and estimation performance.

## 5. Conclusions

In this paper, an improved DOA estimation method with a UCLA is proposed. Exploiting the uniform property of the subarrays, we average the diagonal elements of the two estimated self-covariance matrices to enhance the accuracy of the estimated covariance matrices and the estimation performance. Besides, the polynomial root finding method is utilized instead of exhaustive searching to reduce the computational complexity. The simulation results show that the proposed method can achieve higher resolution and better estimation performance with lower computational complexity.

## Abbreviations

DOA	Direction-of-arrival
UCLA	Unfolded coprime linear array
DOF	Degree of freedom
MUSIC	Multiple Signal Classification
ESPRIT	Estimation of Signal Parameters via Rotational Invariance Techniques
ULA	Uniform linear array
CLA	Coprime linear array
AIC	Akaike Information Criterion
MDL	Minimum Description Length
RMSE	Root mean square error
CRB	Cramér–Rao lower bound
SNR	Signal-to-noise ratio
